# Comprehensive analysis of endoplasmic reticulum stress-associated genes signature of ulcerative colitis

**DOI:** 10.3389/fimmu.2023.1158648

**Published:** 2023-05-23

**Authors:** Beiying Deng, Fei Liao, Yinghui Liu, Pengzhan He, Shuchun Wei, Chuan Liu, Weiguo Dong

**Affiliations:** ^1^ Department of Gastroenterology, Renmin Hospital of Wuhan University, Wuhan, China; ^2^ Department of Geriatric, Renmin Hospital of Wuhan University, Wuhan, China

**Keywords:** endoplasmic reticulum stress, ulcerative colitis, molecular docking, biomarkers, pharmacology

## Abstract

**Background:**

Endoplasmic reticulum stress (ERS) is a critical factor in the development of ulcerative colitis (UC); however, the underlying molecular mechanisms remain unclear. This study aims to identify pivotal molecular mechanisms related to ERS in UC pathogenesis and provide novel therapeutic targets for UC.

**Methods:**

Colon tissue gene expression profiles and clinical information of UC patients and healthy controls were obtained from the Gene Expression Omnibus (GEO) database, and the ERS-related gene set was downloaded from GeneCards for analysis. Weighted gene co-expression network analysis (WGCNA) and differential expression analysis were utilized to identify pivotal modules and genes associated with UC. A consensus clustering algorithm was used to classify UC patients. The CIBERSORT algorithm was employed to evaluate the immune cell infiltration. Gene Set Variation Analysis (GSVA), Gene Ontology (GO), and Kyoto Encyclopedia of Genes and Genomes (KEGG) were used to explore potential biological mechanisms. The external sets were used to validate and identify the relationship of ERS-related genes with biologics. Small molecule compounds were predicted using the Connectivity Map (CMap) database. Molecular docking was performed to simulate the binding conformation of small molecule compounds and key targets.

**Results:**

The study identified 915 differentially expressed genes (DEGs) and 11 ERS-related genes (ERSRGs) from the colonic mucosa of UC patients and healthy controls, and these genes had good diagnostic value and were highly correlated. Five potential small-molecule drugs sharing tubulin inhibitors were identified, including albendazole, fenbendazole, flubendazole, griseofulvin, and noscapine, among which noscapine exhibited the highest correlation with a high binding affinity to the targets. Active UC and 10 ERSRGs were associated with a large number of immune cells, and ERS was also associated with colon mucosal invasion of active UC. Significant differences in gene expression patterns and immune cell infiltration abundance were observed among ERS-related subtypes.

**Conclusion:**

The results suggest that ERS plays a vital role in UC pathogenesis, and noscapine may be a promising therapeutic agent for UC by affecting ERS.

## 1 Introduction

Ulcerative colitis (UC) is a subtype of inflammatory bowel disease (IBD) characterized by alternating relapses and remissions. Although the pathogenesis of UC involves several factors, including host genetic susceptibility, intestinal microbiota, environmental factors, and immunological abnormalities (1), it is not yet fully understood. Despite expanding therapeutic options, treatment of UC remains highly challenging.

Studies have suggested that imbalance of endoplasmic reticulum stress (ERS) in the epithelial cells of the intestine contributes to the progress of IBD (2). In particular, large amounts of ERS have been observed in the intestinal epithelium of UC (3). ERS is associated with the development of fibrosis in patients with Crohn’s disease (CD) (4). Prolonged and sustained ERS promotes inflammation, increases the production of reactive oxygen species, enhances M1 macrophage polarization, and disrupts the epithelial barrier (5). Moreover, unresolved ERS can instigate small intestine inflammation (6).

ER is primarily responsible for facilitating proper protein folding and translocation to corresponding functional destinations (7) and is susceptible to changes in intracellular homeostasis and extracellular stimuli. Inflammatory responses can impair ER function and lead to accumulated overload of unfolded and misfolded proteins, resulting in ERS (8). The unfolded protein response (UPR) is activated to mitigate ERS-induced cellular damage and enhance cellular resistance to injury. UPR signaling is primarily mediated by IRE1, PERK, and ATF6, which maintain ER homeostasis by upregulating ERS protein expression, inhibiting translation, reducing protein synthesis, and upregulating the expression of genes encoding ER-related degradation effects. However, when ERS is too intense, the UPR may not fully compensate for cellular damage, leading to apoptotic signaling pathway activation and cell removal to reduce tissue damage (2). However, little is known about its ERS-related molecular mechanisms in UC.

We identified pivotal ERS-related molecular mechanisms and their relationship with biologics in UC pathogenesis. In addition, we conducted immune infiltration, consensus clustering, and molecular docking analyses to describe the impact of ERS on UC. The understanding of the relationship between UC and ERS is expected to contribute to the comprehension of mechanisms and provide a new perspective on therapeutic strategies for UC.

## 2 Materials and methods

### 2.1 Data acquisition and pre-processing

Gene expression data and matched clinical information were retrieved from the Gene Expression Omnibus (GEO) database (http://www.ncbi.nlm.nih.gov/geo). Details of datasets are listed in **Table 1**. We included a total of 1,350 ERGs with correlation scores >5 from the GeneCards database (https://www.genecards.org/), which are available in **Supplementary Material S1**.

**Table 1:** The information of all the datasets in the study.

Dataset	Platform	Title
GSE87466	GPL13158	[HT_HG-U133_Plus_PM] Affymetrix HT HG-U133 + PM Array Plate
GSE206285	GPL13158	[HT_HG-U133_Plus_PM] Affymetrix HT HG-U133+ PM Array Plate
GSE107499	GPL15207	[PrimeView] Affymetrix Human Gene Expression Array
GSE179728	GPL16791	Illumina HiSeq 2500 (*Homo sapiens*)
GSE73661	GPL6244	[HuGene-1_0-st] Affymetrix Human Gene 1.0 ST Array
GSE92415	GPL13158	[HT_HG-U133_Plus_PM] Affymetrix HT HG-U133 + PM Array Plate

### 2.2 Assessment of immune infiltration patterns in UC

The CIBERSORT algorithm was used to evaluate the abundance of 22 immune cell types in the colon mucosa of UC patients and healthy controls (9). Student’s t-test was applied to verify the differences between the two groups, and results were presented using the “ggboxplot” R package. The relationship between ERSRGs and immune cells was evaluated with the “corrplot” R package, and the results were presented using the “pheatmap” R package.

### 2.3 Differential expression gene analysis and functional analysis

The “limma” R package was used to detect differentially expressed genes (DEGs) using p<0.01 and |log2 FC|>1 as threshold values (10). The results were visualized using “ggplot2” and “pheatmap” R packages. The R packages “clusterProfiler” and “org.Hs.eg.db” were used to determine the underlying biological mechanisms of identified DEGs (11). Gene Ontology (GO) and Kyoto Encyclopedia of Genes and Genomes (KEGG) enrichment analyses were performed to determine statistically significant enrichment using *q*-value < 0.05 as a threshold.

### 2.4 Weighted gene co-expression network analysis

We performed weighted gene co-expression network analysis (WGCNA) analysis using the WGCNA R package, which builds scale-free co-expression networks for clinical phenotypes (12). Initially, hierarchical clustering analysis was conducted to filter discrete cases. Subsequently, an appropriate soft power β was selected for weighted adjacency matrix construction, which was transformed into a topological overlap matrix (TOM) containing module assignments that were labeled by color and module feature (ME). Additionally, Pearson correlation coefficients were calculated to evaluate the correlation between ME and clinical characteristics.

### 2.5 Identification of ERS-related genes

Hub genes were extracted by screening for genes from the module with the highest relevance to UC in WGCNA according to the criteria of gene significance (GS) >0.25 and module membership (MM) >0.7 (13). ERS-related genes (ERSRGs) were obtained by intersecting hub genes, ERGs, and DEGs using a Venn diagram. Finally, ROC analysis was performed using the “pROC” R package to assess the efficacy of ERSRGs to diagnose UC.

To study the interplay among ERSRGs, a protein interaction (PPI) network was constructed using the STRING database (version: 11.5, http://string-db.org/) (14). After filtering out disconnected nodes, the network was imported into Cytoscape software (version: 3.8.2, https://cytoscape.org) for visualization (15). Significant gene clusters were identified using the MCODE and cytohubba-MCC plugins (16). Cluster genes with a score >10 were subsequently visualized. The iRegulon plugin was used to identify key transcription factors (17).

### 2.6 Classification of ERS-related molecular subtypes and functional enrichment analysis

To assess the biological functions of ERSRGs in UC, we conducted an unsupervised consensus clustering analysis based on ERSRGs expression using the “ConsensusClusterPlus” R package (18). Principal component analysis (PCA) was then used to verify the patterns of gene expression in the distinct clusters.

Two gene sets (“c2.cp.kegg.v7.4.symbols” and “c5.go.bp.v7.5.1. symbols”) were downloaded from the MSigDB database (https://www.gsea-msigdb.org/gsea/msigdb/) as input files for Gene Set Variation Analysis (GSVA) (19). GSVA scores were calculated between different ERS-related subtypes using the R package “limma” to identify differentially enriched functions and pathways. Functions and pathways with GSVA scores with |t-values| >2 were identified as significantly enriched.

### 2.7 Small molecule agents screening and molecular-ligand docking analysis

The connectivity map database (CMap, https://clue.io/) is a differential gene expression-based drug prediction database, which is primarily used to explore the functional relationships among genes, small molecule compounds, and diseases (20, 21). The primary protein structures of the target genes were downloaded from The Protein Data Bank database (http://www.rcsb.org, PDB). AutoDock Tools software (version 1.5.7) was used to molecularly dock the key targets to small molecule compounds (22). The binding activities of small molecule compounds and targets were evaluated based on docking energy values using Pymol software (http://www.pymol.org).

### 2.8 Statistical analysis

All statistical analyses were performed using R software (version 4.2.1, https://www.r-project.org) and associated R packages. All data were expressed as mean ± SE. Comparisons between two groups were performed using the t-test, and comparisons among three or more groups were performed using one-way ANOVA. Correlation analysis was performed using Spearman’s correlation analyses using the R software packages “ggpubr” and “stats,” and *p*<0.05 was considered statistically significant.

## 3 Results

### 3.1 Identification of DEGs and functional annotation and pathway enrichment of DEGs

**Figure 1:**
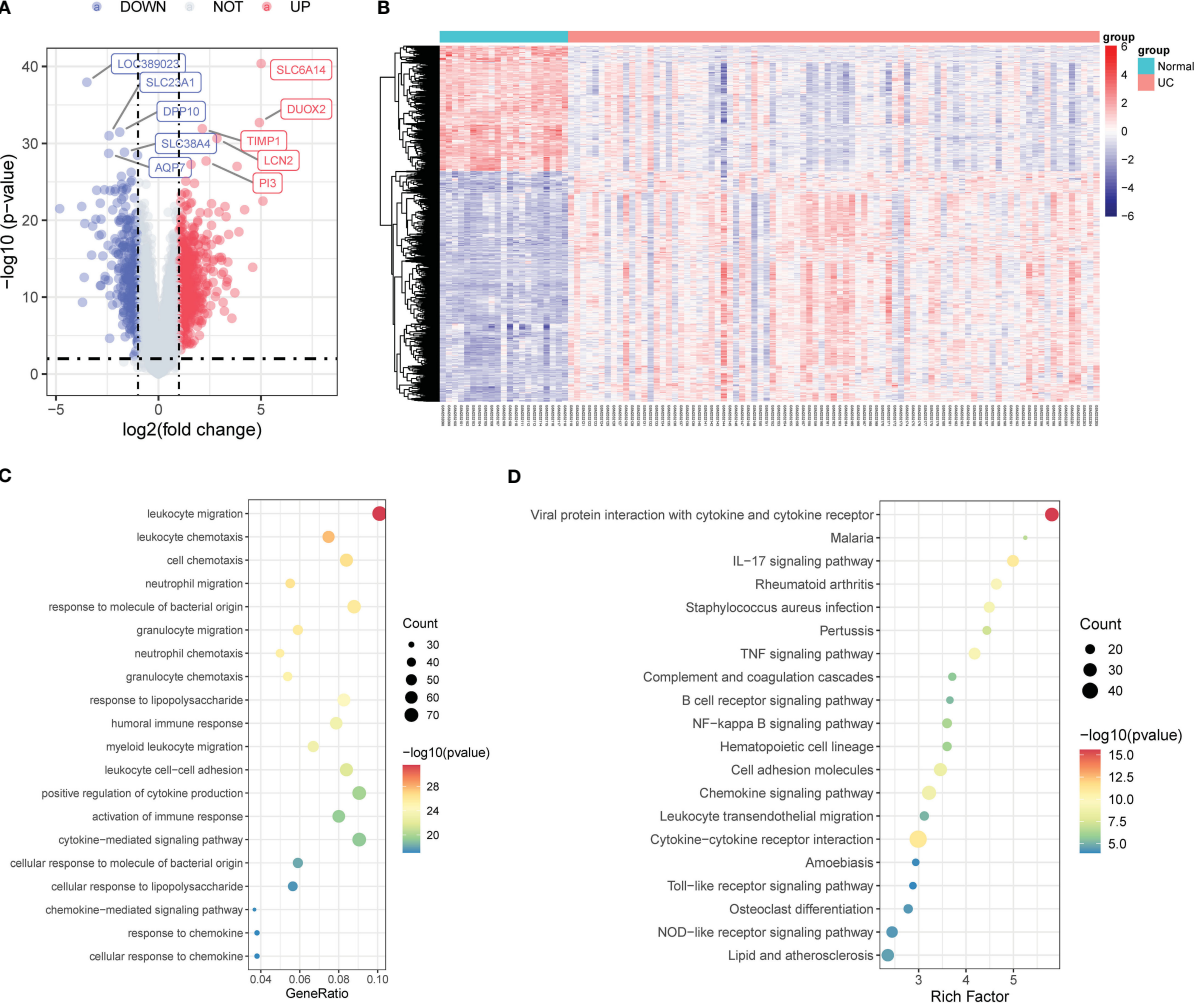
Identification of DEGs associated with UC. **(A)** Volcano map of DEGs in the colonic mucosal between UC patients and healthy controls. **(B)** The expression profiles of DEGs between UC patients and healthy controls. **(C)** GO enrichment analysis of DEGs. **(D)** KEGG pathway enrichment analysis of DEGs. DEGs, differentially expressed genes; UC, ulcerative colitis; GO, the Gene Ontology; KEGG, the Kyoto Encyclopedia of Genes and Genomes analyses.

We utilized the “limma” package to analyze the DEGs in the colonic mucosa of UC patients and healthy controls and identified 915 DEGs using *p*-value <0.01 and |log_2_FC|>1 as thresholds, among which 593 were upregulated and 322 were downregulated (**Figure 1A**). The heatmap demonstrated the expression pattern of DEGs and the relative consistency within the groups (**Figure 1B**).

Furthermore, we performed pathway enrichment analyses on the 915 DEGs to better understand the latent mechanisms and functions in UC. GO enrichment analysis results indicated significant involvement of these genes in signaling pathways such as leukocyte migration, leukocyte chemotaxis, and neutrophil migration (**Figure 1C**). The KEGG enrichment analysis results revealed the activation of multiple inflammatory response-related pathways, including the IL-17 signaling pathway, tumor necrosis factor (TNF) signaling pathway, B-cell receptor signaling pathway, nuclear factor kappa B (NF-κB) signaling pathway, chemokine signaling pathway, cytokine–cytokine receptor interaction, Toll-like receptor signaling pathway, and NOD-like receptor signaling pathway (**Figure 1D**).

### 3.2 Identification of ERSRGs

**Figure 2:**
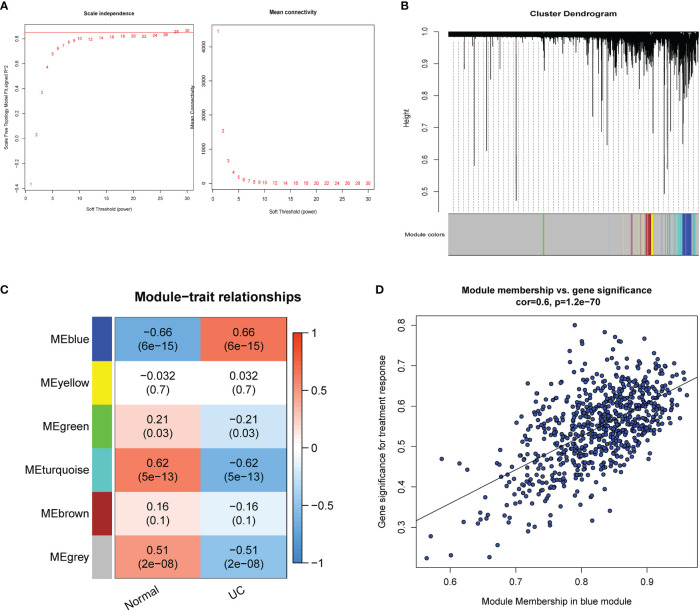
Identification of modules associated with UC. **(A)** Scale-free index analysis for soft-threshold power and mean connectivity analysis for various soft-threshold powers. **(B)** Module clustering dendrogram based on a dissimilarity measure (1-TOM). **(C)** Heatmap of the correlation between module eigengenes and UC. **(D)** Scatter plot of the GS for the UC vs. the MM in the blue modules. TOM, topological overlap matrix; GS, gene significance; MM, module membership.

Using the WGCNA for module classification, we identified six modules (**Figures 2A, B**). Based on the correlation coefficient between modules and UC, we selected the blue module (r=0.66, p=6e−15) with the highest correlation with UC as the key module (**Figure 2C**). We screened 680 hub genes from the blue module for subsequent analysis, based on GS >0.25 and MM >0.7 (**Figure 2D**).

**Figure 3:**
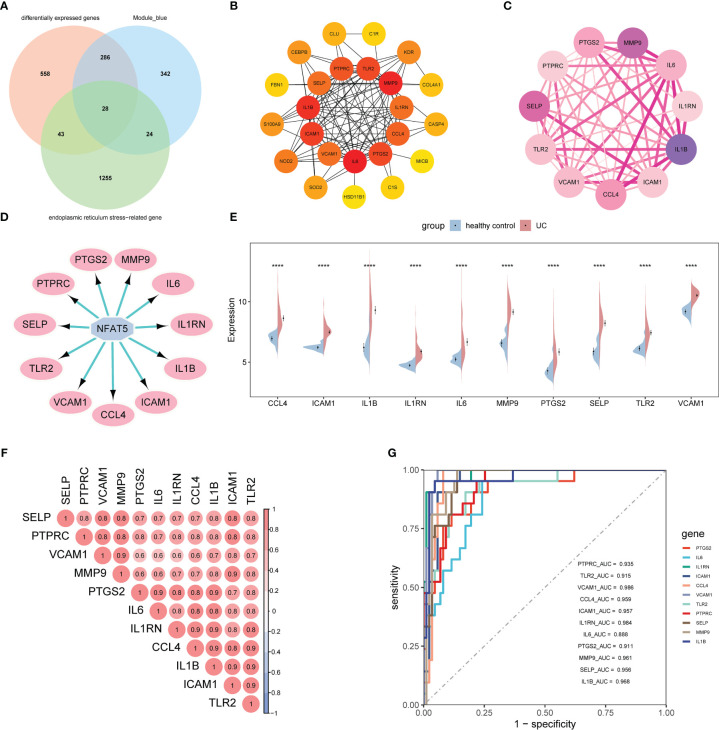
Identification of ERSRGs. **(A)** Venn diagram showed the overlapping genes between differentially expressed genes, ERGs, and the genes of the blue module. **(B)** Cytohubba-MCC was used to identify hub genes in the protein interaction network of ERSRGs. **(C)** MCODE sub-network. **(D)** The master regulator is predicted by the iRegulon tool. The pink node represents the regulator and target genes are highlighted in blue. **(E)** Split violin plot revealing the expressional differences in ERSRGs between UC patients and healthy control in the GSE87466 dataset. **(F)** Correlation of ERSRGs. **(G)** ROC curves of ERSRGs in the GSE87466 dataset. ERGs, endoplasmic reticulum stress genes; ERSRGs, endoplasmic reticulum stress-related genes; ROC, receiver operating characteristic. ****p < 0.0001.

To narrow down the ERSRGs, we obtained 28 genes via Venn diagrams by identifying genes common to ERGs, DEGs, and hub genes (**Figure 3A**). To identify their interactions, we constructed a PPI network using the STRING database and identified 11 genes with a degree >10, using the cytoHubba-MCC plugin (**Figure 3B**). Subsequently, we identified two cluster modules using the MCODE plugin, with cluster 1 having a higher score (score of 10.6000, 11 nodes, and 53 edges), consistent with the result of cytoHubba-MCC analysis (**Figure 3C**). We used the iRegulon plugin to test the transcription factor (TF) binding patterns of 11 genes, which showed that all genes are regulated by NFAT5 (**Figure 3D**).

**Figure 3E** showed the expression patterns of the 11 ERSRGs between the UC group and healthy controls. We also analyzed the correlations among ERSRGs and found significant synergistic effects (**Figure 3F**). Subsequently, we performed ROC analysis to verify the diagnostic significance of ERSRGs (**Figure 3G**). All genes had area under the curve (AUC) values >0.75, with VCAM1 having the largest AUC value (AUC, 0.986) and IL-6 the smallest (AUC, 0.888).

### 3.3 Prediction of potential therapeutic drugs for UC patients

**Figure 4:**
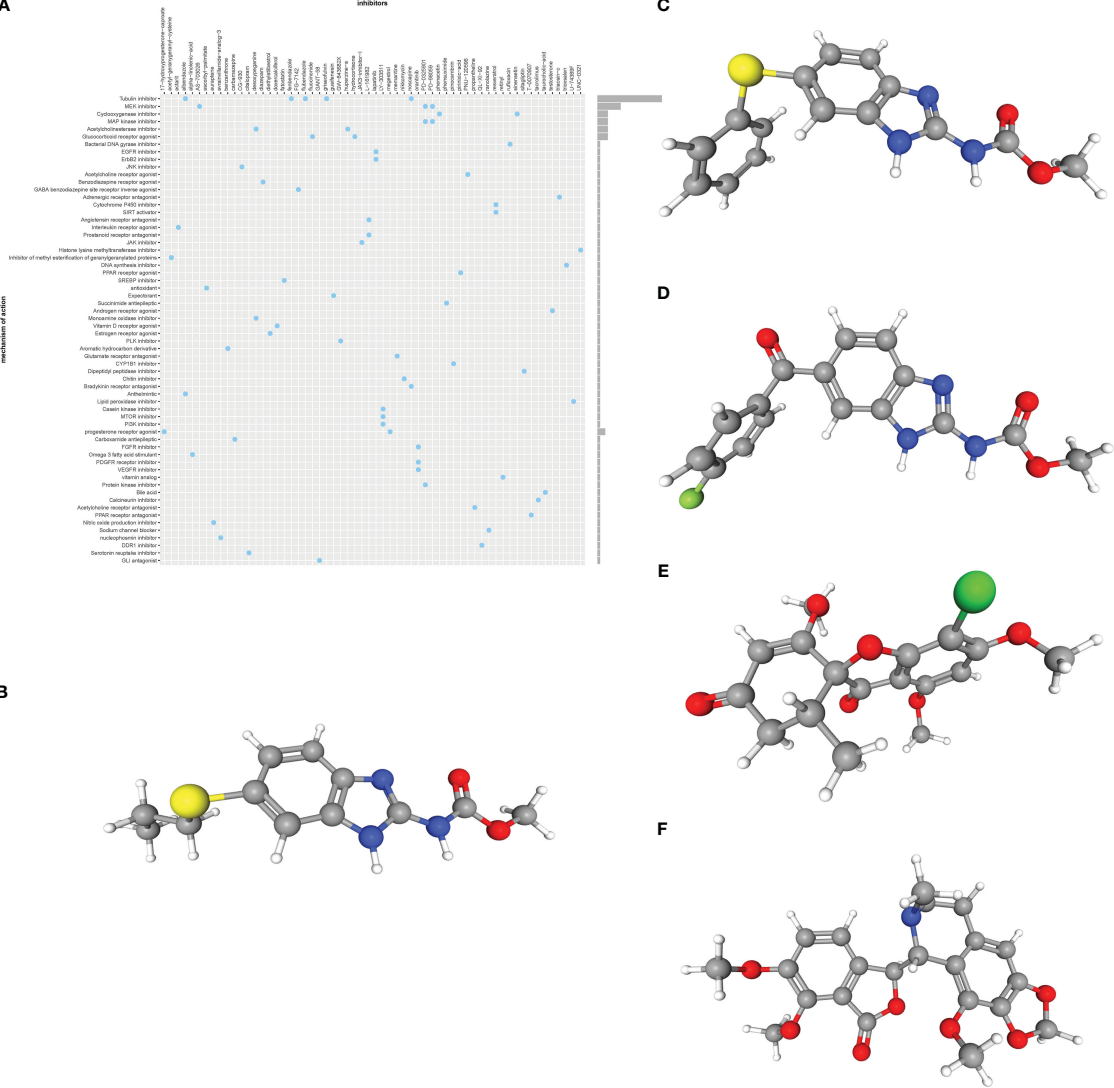
**(A)** CMap analysis showed the mechanism of action based on small-molecule compounds. **(B–F)** 3D structures of small molecule drugs predicted using the PubChem open chemical database, including albendazole **(B)**, fenbendazole **(C)**, flubendazole **(D)**, griseofulvin **(E)**, and noscapine **(F)**. CMap, the Connectivity Map.

We submitted the 11 ERSRGs to the CMap database to screen for promising small molecule compounds that could be used for UC management. Using this approach, we identified five potential small-molecule drugs that shared tubulin inhibitory effects: albendazole, fenbendazole, flubendazole, griseofulvin, and noscapine (**Figure 4A**). The structures of these compounds were retrieved from the PubChem database and are displayed in **Figures 4B–F**. Based on the correlation scores between drugs and genes, noscapine was selected for subsequent analysis.

### 3.4 The molecular docking landscape on UC drugs against ERS

**Figure 5:**
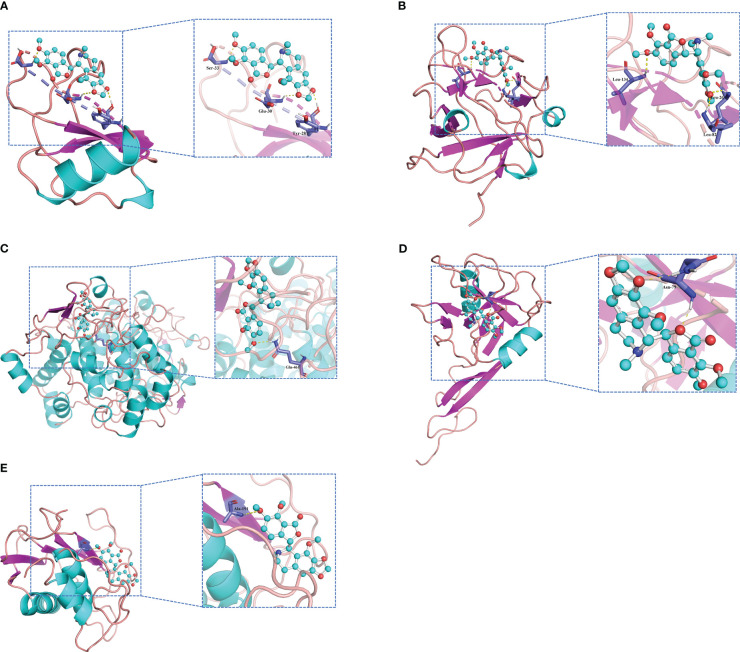
Molecular docking pattern of noscapine complexed with CCL4 **(A)**, IL1B **(B)**, PTGS2 **(C)**, SELP **(D)**, and MMP9 **(E)**.

Molecular docking is an important method for structure-based drug design and screening by finding the optimal conformation of small molecule compounds and target molecules for interaction. In this study, the crystal structures of five molecular targets with high resolutions (smaller than 3 Å), i.e., CCL4 (PDB ID: 3TN2), IL1B (PDB ID: 5R8Q), MMP9 (PDB ID: 6ESM), SELP (PDB ID: 1G1Q), and PTGS2 (PDB ID: 5F19), were downloaded from the RCSB Protein Data Bank. We used AutoDock Tools1.57 software to dock noscapine and the five molecular targets with the largest fold difference. The docking scores were less than −6 kcal/mol, suggesting a high binding affinity of noscapine with the targets. The binding poses and sites are shown in **Figure 5**, where the red color represents the compounds, and the yellow dotted lines represent hydrogen bond interactions.

### 3.5 Verification of ERSRGs

**Figure 6:**
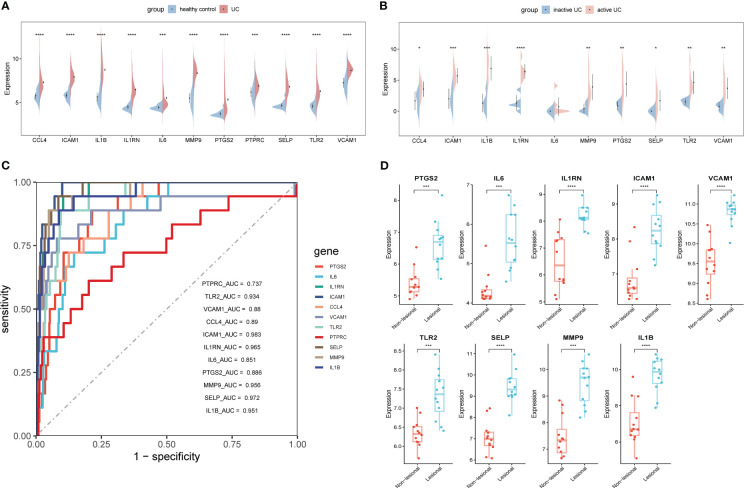
External validation of ERSRGs **(A, B)**. **(A)** Split violin plot revealing the expression differences in ERSRGs between UC patients and healthy controls in the GSE206285 dataset. **(B)** ROC curves of ERSRGs in the GSE206285 dataset. **(C)** Split violin plots revealing the expressional differences in ERSRGs between inactive and active UC patients. **(D)** The expression of ERSRGs in the lesional and non-lesional colonic tissues from active UC patients in the GSE107499 dataset. *p < 0.05, **p < 0.01, ***p < 0.001, ****p < 0.0001.

To validate the expression levels of 11 ERSRGs, we used an external dataset (GSE206285). The expression levels of ERSRGs in the colon tissue of UC patients were significantly higher compared to healthy controls (**Figure 6A**). ROC analysis showed that, except for PTPRC, the AUCs of all ERSRGs were >0.750 (**Figure 6B**). Based on the ROC analysis result, we further analyzed 10 ERSRGs (excluding PTPRC). The levels of these genes were found to be higher in active UC patients compared to inactive UC patients in the GSE179128 dataset, except for IL-6 (**Figure 6C**). We also analyzed the GSE107499 dataset, which contains inflammatory and non-inflammatory colonic tissues from active UC patients. CCL4 was not detected in this dataset; hence, we validated the expression of the other nine ERSRGs, which were found to be upregulated in diseased colonic tissues of active UC patients (**Figure 6D**).

### 3.6 Immune-infiltrating landscape of active UC

**Figure 7:**
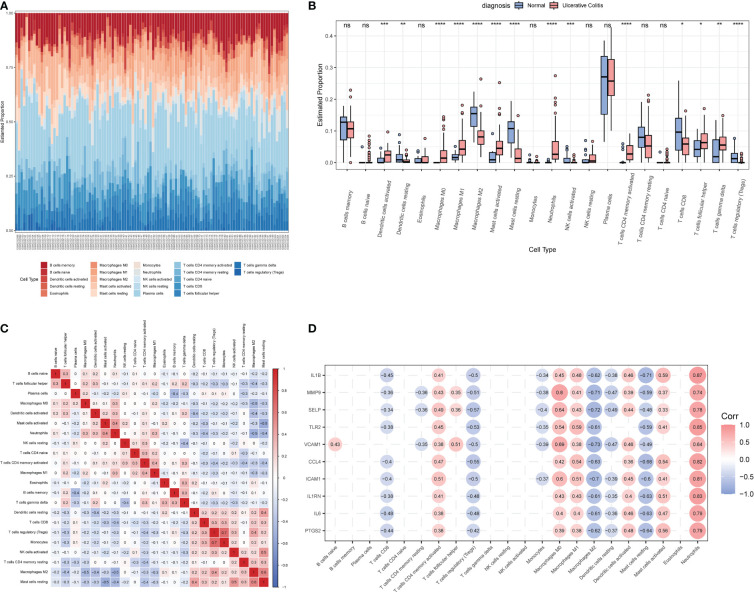
The immune characteristics between the UC group and healthy controls in the GSE206285 dataset. **(A)** Histogram showing the distribution of 22 immune cells infiltration between the UC group and healthy control. **(B)** Boxplot showing the differences in infiltrated immune cells between the UC group and healthy controls. **(C)** Heatmap delineating the correlation in immune cell infiltrations. **(D)** Correlation of ERSRGs and 22 immune cell types. *p < 0.05, **p < 0.01, ***p < 0.001, ****p < 0.0001; ns, not statistically significant.

The CIBERSORT algorithm was employed to characterize the abundance of 22 immune cell infiltrates in the colon tissue of the two groups. **Figure 7A** displays the distribution of immune cell types in each sample for both groups, while **Figure 7B** illustrates the differences in the abundance of infiltrated immune cells between the two groups. In comparison to healthy controls, higher levels of activated dendritic cells, M0 and M1 macrophages, activated mast cells, neutrophils, activated CD4+ memory T cells, follicular helper T cells, and γ&δ T cells were found to be infiltrating the colonic mucosa of UC patients. **Figure 7C** depicts the correlation between the 22 immune cell types, with Tregs and monocytes displaying the highest correlation (*r* = 0.7).

Next, the correlations between 10 ERSRGs and immune cells (**Figure 7D**) were examined. The results indicated that naive B cells, CD4 memory-activated T cells, follicular helper T cells, M0 and M1 macrophages, activated dendritic cells, γ&δ T cells, activated mast cells, and neutrophils were positively correlated with the 10 ERSRGs, with neutrophils exhibiting the strongest correlation.

### 3.7 Colonic mucosal invasion of active UC is associated with the ERS

**Figure 8:**
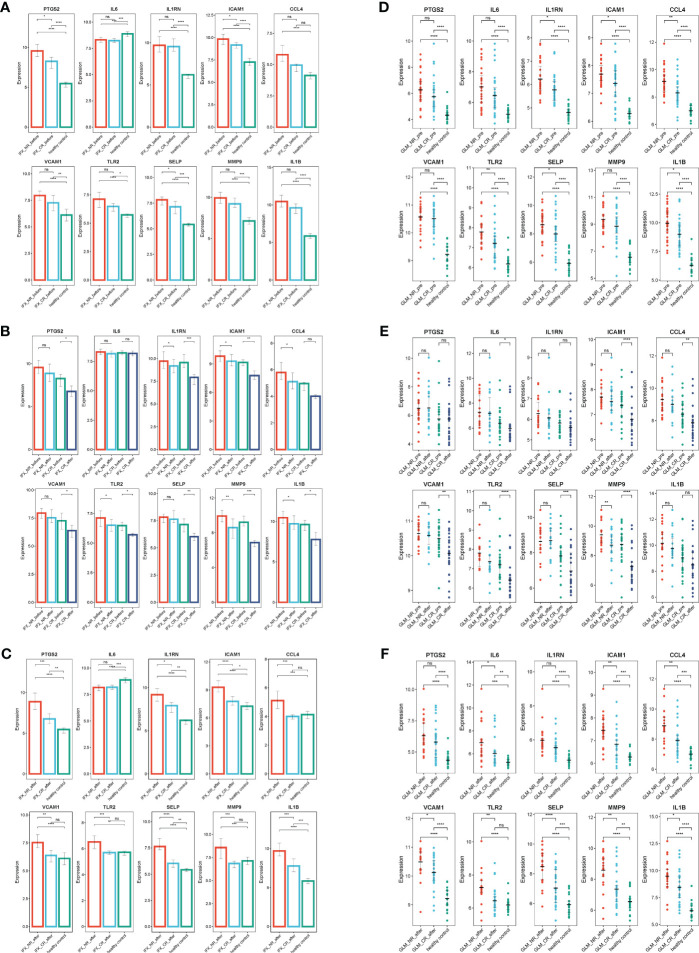
IFX and GLM responders improve impaired colonic mucosal in UC patients by regulating the ERSRGs. **(A–C)** The relative expression levels of ERSRGs in the colonic mucosa of healthy controls, UC patients in not responding and responding groups before and after IFX therapy. **(D–F)** The relative expression levels of ERSRGs in the colonic mucosal of healthy controls, UC patients in responding and non-responding groups before and after GLM treatment. IFX, infliximab; GLM, golimumab. *p < 0.05, **p < 0.01, ***p < 0.001, ****p < 0.0001, ns, not statistically significant.

Biologics such as TNF-α inhibitors infliximab (IFX), golimumab (GLM), and IL-12/IL-23 inhibitors ustekinumab (Ust) are approved for the treatment of UC and are proposed as first-line treatment for moderate to severe UC. The effects of biologics on ERS were explored using the GSE73661, GSE92415, and GSE206285 datasets. The results indicated that, before IFX treatment, ERSRGs in UC patients were significantly elevated compared to healthy controls, except for IL-6 and CCL4. In the clinical response group, the expression levels of PTGS2, ICAM1, and SELP were significantly reduced compared to the non-response group (**Figure 8A**). In the IFX clinical response group, the expression of ERSRGs, with the exception of IL-6 and CCL4, was significantly reduced after IFX treatment (**Figure 8B**). Importantly, VCAM1, TLR2, and MMP9 expression levels in the clinical response group returned to those of healthy controls after IFX treatment (**Figure 8C**).

GSE92415 contains expression profiles of biopsy samples from UC patients treated with GLM. Before GLM treatment, the expression of all 10 ERSRGs in active UC patients was higher compared to healthy controls, with significantly lower expression levels of IL1RN, ICAM1, CCL4, TLR2, SELP, and IL1B in the clinical response group than in the non-responder group (**Figure 8D**). After GLM treatment, the expression of IL-6, ICAM1, CCL4, VCAM1, TLR2, SELP, and MMP9 was reduced (*p*< 0.05) in the clinical response group (**Figure 8E**). However, only TLR2 expression levels returned to those of healthy controls (**Figure 8F**).

**Figure 9:**
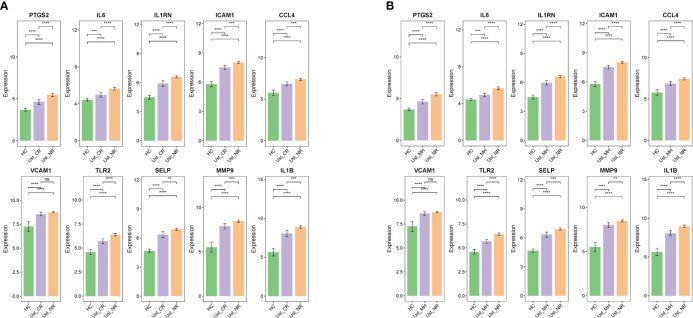
Ust reduces impaired colonic mucosal in UC patients by regulating the ERSRGs **(A, B)**. The relative expression levels of ERSRGs in the colonic mucosal of HC (healthy control), Ust_CR (UC patients in remission before Ust therapy), Ust_NR (UC patients not responding before Ust therapy), and Ust_MH (UC patients in mucosal healing before Ust therapy). Ust, ustekinumab; CR, clinical remission; MH, mucosal healing. **p < 0.01, ***p < 0.001, ****p < 0.0001, ns, not statistically significant.

Finally, GSE206285 contains expression profiles of baseline biopsy samples from Ust-treated patients with moderate to severe UC. Prior to Ust treatment, ERSRGs in the clinical response group were significantly reduced compared to the non-response group, except for VCAM1 (**Figure 9A**). Additionally, the dataset assessed mucosal healing in UC patients treated with Ust. We found that baseline expression patterns of ERSRGs in mucosal healing patients were similar to those of clinical responders (**Figure 9B**).

### 3.8 Identification of ERS-related subtypes in UC

**Figure 10:**
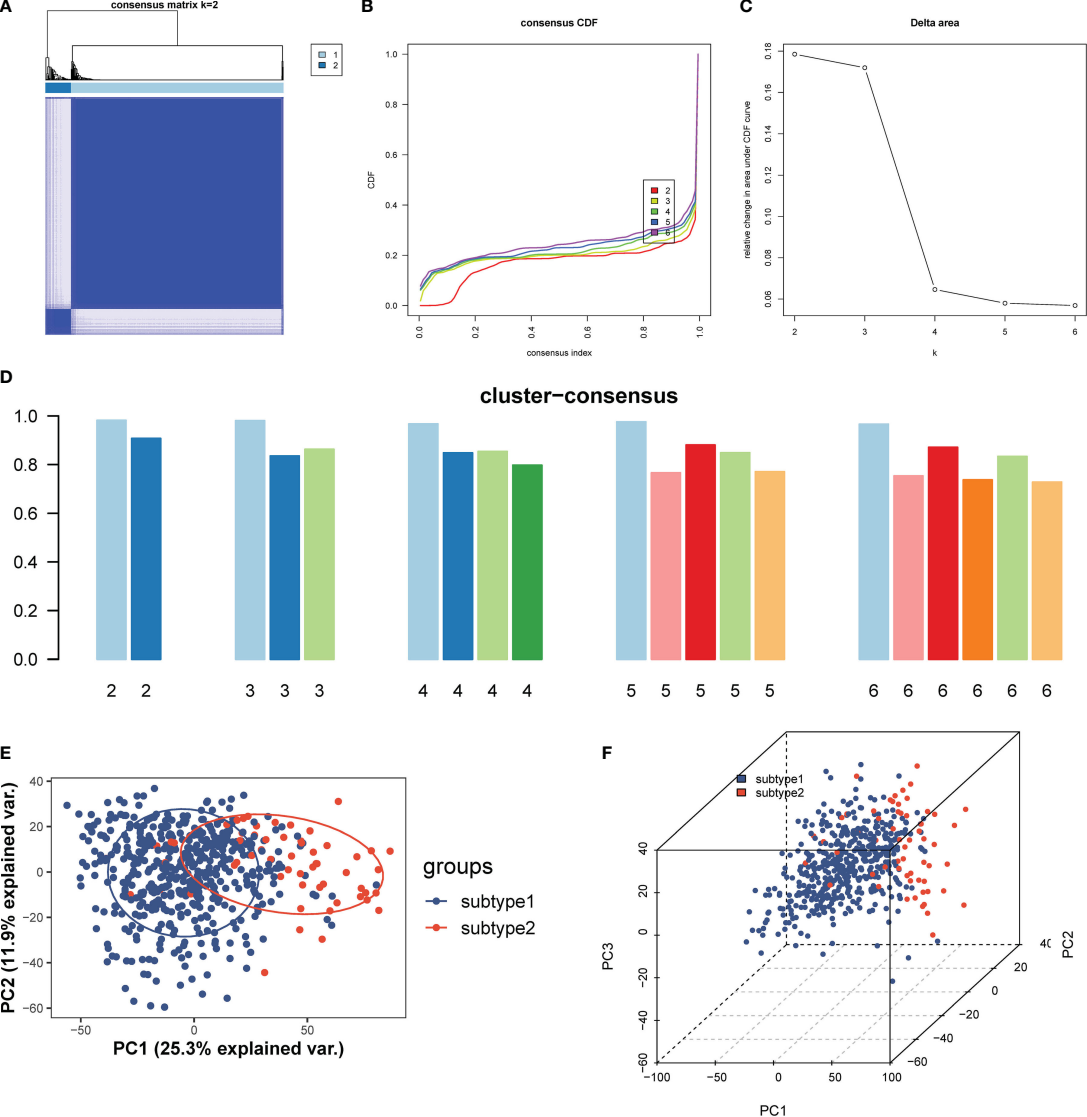
Identification and enrichment analysis of ERS-related subtypes. **(A)** Consensus clustering matrix when k = 2. **(B)** Consensus CDF curves when k=2 to 6. **(C)** Relative alterations in CDF delta area curves. **(D)** Consensus score of each subtype when k=2 to 6. **(E, F)** PCA diagram separated subtype1 (blue) and sybtype2 samples (red). CDF, cumulative distribution function; PCA, principal component analysis.

To illustrate the ERS-related patterns of UC, we performed an unsupervised cluster analysis on 550 UC samples from the GSE206285 dataset using the “ConsensusClusterPlus” R package based on the expression patterns of 10 ERSRGs. We observed stable isoform numbers when k=2 (**Figures 10A, B**), and significant differences in the relative changes in the area under the CDF curve from k=2 to k=6 (**Figure 10C**). The consistency scores of the subtypes were highest when k=2 (all over 0.8) (**Figure 10D**). Therefore, we divided the 550 UC samples into two subtypes, namely, subtype 1 (n = 491) and subtype 2 (n = 59), based on the significant differences observed using PCA analysis (**Figures 10E, F**).

**Figure 11:**
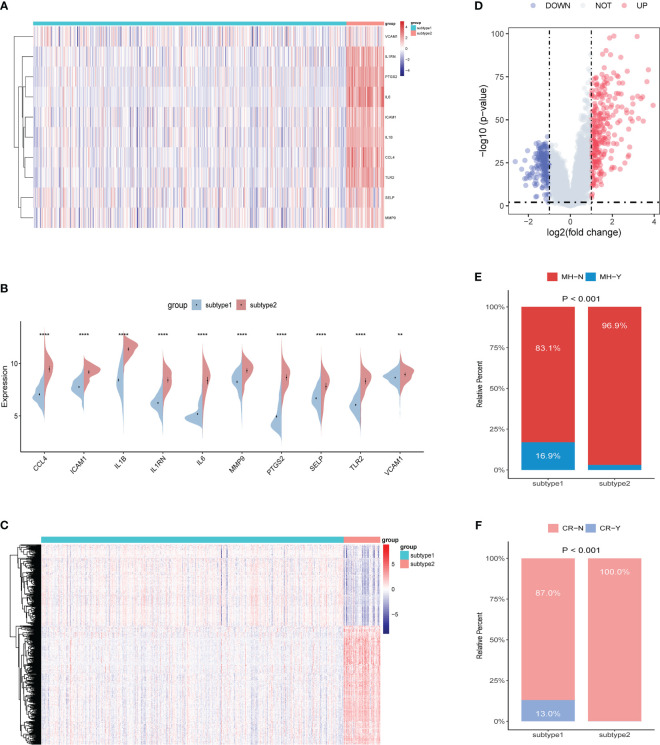
Identification of the differentiation of feature genes between ERS-related subtypes. **(A, B)** Heatmap **(A)** and split violin plot **(B)** revealing the expression of 10 characteristic genes between subtypes. **(C, D)** Heatmap **(C)** and volcano map **(D)** revealing the gene expression patterns between the two subtypes. **(E, F)** Rate of mucosal healing **(E)** and clinical response **(F)** in the subtype1 and subtype2 UC patients after undergoing a duration of 8 weeks of Ust treatment.

To better understand the molecular characteristics between subtypes, we evaluated the differences in the expression of 10 ERSRGs. The results showed that all 10 ERSRGs were significantly elevated in subtype 2 (**Figures 11A, B**). Further analysis revealed significant differences in gene expression patterns between the two subtypes (**Figures 11C, D**).

To evaluate the disparities in the impact of Ust treatment between the two distinct subtypes of UC patients (subtypes 1 and 2), we performed a comparative analysis. The results showed that the percentage of mucosal healing and clinical response were remarkably higher in the subtype1 group of UC patients after undergoing a duration of 8 weeks of Ust treatment (**Figures 11E, F**).

**Figure 12:**
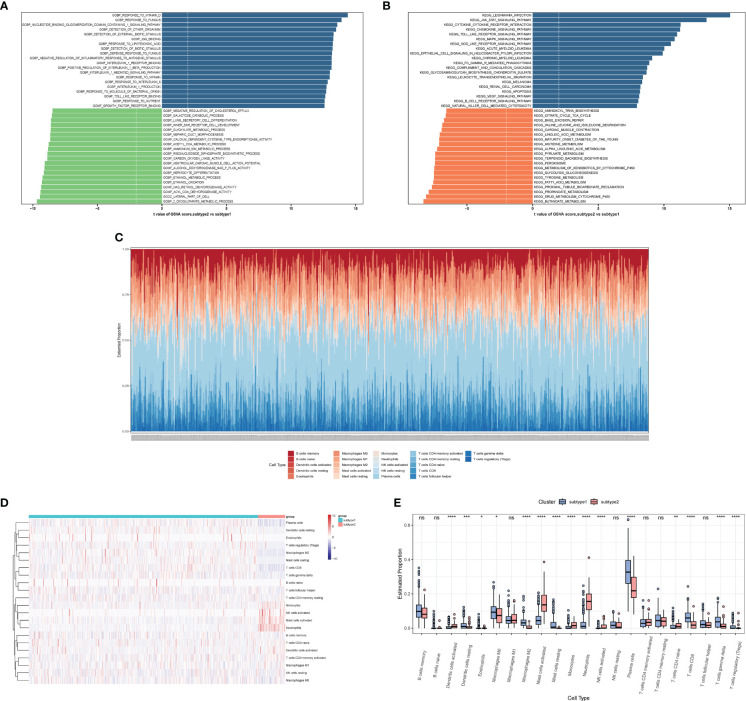
The immune characteristics of distinct ERS-related subtypes. **(A, B)** Differences in enriched biological functions **(A)** and hallmark pathways **(B)** between distinct immune microenvironment subtypes ranked by t values of GSVA scores. **(C)** Histogram showing the distribution of infiltration of 22 immune cell types between subtypes. **(D)** Heatmap delineating the abundance of various infiltrated immune cell types between subtypes. **(E)** Boxplot showing the differences of infiltrated immune cells between subtypes. GSVA, Gene Set Variation Analysis.

We performed a GSVA analysis to assess differences in functions and pathways enriched in ERS-related subtypes. The analysis revealed that various pathways, including IL1β production, IL1 production, IL1 receptor binding, response to IL-6, IL-1-mediated signaling pathways, Toll-like receptor binding, and growth factor receptor binding were upregulated in subtype 2 (**Figure 12A**). In addition, FCγR-mediated phagocytosis, apoptosis, and cytokine-related pathways were upregulated in subtype 2 (**Figure 12B**). Moreover, B-cell receptor signaling pathways, NK-cell-mediated cytotoxicity, JAK/STAT signaling pathway, Toll-like receptor signaling pathway, MAPK signaling pathway, NOD-like receptor signaling pathways, and VEGF signaling pathways were also enriched in subtype 2.

We also observed differences in the abundance of immune cell infiltration between subtype 1 and subtype 2 (**Figures 12C–E**). Subtype 1 showed more resting dendritic cells, M0 macrophages, M2 macrophages, resting mast cells, plasma cells, CD8 T cells, γ&δ T cells, and Tregs. Subtype 2 showed more activated dendritic cells, activated mast cells, monocytes, neutrophils, activated NK cells, and naive CD4 T cells.

## 4 Discussion

Long-term and persistent ERS can affect the secretory function of Paneth cells and goblet cells, triggering pathological changes such as an inflammatory cascade response, apoptotic pathways, and mucosal barrier disorders. Modulating ERS could lead to the development of new therapies for treating active UC. In this study, we identified ERSRGs through integrated bioinformatic analysis and found significant synergistic effects among them through correlation analysis.

Noscapine is a phthalate isoquinoline alkaloid that binds to bitter receptors and antagonizes the bradykinin and histaminergic systems (23). It can protect oligodendrocytes from ischemia and chemical injury. A brominated analog of noscapine was shown to inhibit the release of TLRs, TNF-α, and NO from macrophages and alleviate experimental colitis by reducing MPO, IL-1β, and IL-6 (24). In addition, noscapine can inhibit the NF-κB signaling pathway by blocking the phosphorylation and degradation of IκBα via blocking IκB kinase and by blocking the phosphorylation and nuclear translocation of p65 (25). Methyl-noscapine has been shown to alleviate anxiety and depression by blocking small conductance SK channels (26). Molecular docking results suggest that noscapine may be a target agent for UC by affecting ERS, but this specific mechanism requires experimental verification.

Abnormal immune response is considered one of the potential pathogenic mechanisms of UC. We found a significant correlation between upregulated immune cells and ERSRGs. The ERS-mediated apoptotic pathway may be a novel therapeutic target for targeting immune cell apoptosis during UC. We used an unsupervised clustering approach to estimate the molecular pattern of UC colon tissue based on the expression of ERSRGs and ultimately identified two distinct molecular subtypes. The results of enrichment analysis indicated that subtype 2 is tightly involved with differentiation, migration, and activation of immune cells.

We validated the expression pattern of ERSRGs within the UC group versus healthy controls and the lesion versus non-lesion groups using different datasets, all of which showed significant differences, suggesting a key role of ERS in UC progression. Of the UC patients, 80%–90% have alternating active and inactive phases, and ERSRGs expression differed significantly between active and inactive UC. ERS-induced intestinal mucosal damage and increased inflammatory cytokines in intestinal epithelial cells may be important causes of disease recurrence. Focusing on attenuating ERS may be a new way to avoid UC recurrence.

The role of ERS in UC development was investigated by exploring the effect of biological agents on ERSRGs. Significant reductions in ERSRG expression were observed following treatment with biological agents such as IFX, GLM, and Ust, supporting the critical involvement of ERS in UC progression. Further investigation into the link between ERS and UC development is warranted.

Inflammatory cytokines, such as IL-1β and IL-6, have been shown to increase intestinal epithelial permeability and recruit neutrophils to inflamed colonic tissue, leading to mucosal edema and necrosis and disrupting the intestinal epithelial barrier (27, 28). Increased production of inflammatory cytokines, such as IL-1β and IL-6, has a pivotal role in activating ERS (29–31). Conversely, ERS promotes secretion of IL-1, IL-6, and other pro-inflammatory cytokines (32). Targeting IL-1β has shown promise in alleviating UC (33), and treatment with 50 mg of humanized anti-IL-6 mAb had significantly higher response ratios at weeks 8 and 12 for patients with CD (34).

Inflammatory cytokines can regulate prostaglandin synthesis by inducing expression through the TLR/NF-κB pathway in macrophages and epithelial cells. PTGS2/COX-2 deletion in circulating/resident myeloid cells increases susceptibility to DSS-induced colitis (35). COX-2’s role in mediating barrier dysfunction has been examined in mice and has been implicated in facilitating colonic infection. Excessive expression of COX-2 has also been observed in the epithelium of the colon in UC patients (36), and ERS has been shown to regulate COX-2 expression through the NF-κB pathway (37). Furthermore, ERS has been shown to promote the expression of COX-2 via eIF2α-ATF4 and mediate autophagy (38). In addition, COX-2 can also activate ERS via the BIP/CHOP pathway and exacerbate lung injury (39). Celecoxib inhibits the COX-2-mediated PI3K/Akt pathway and reduces ERS in hepatocellular carcinoma cells (40).

The inflammatory response is linked to TLR-mediated activation of the ERS (41). TLR2 is partially localized to the ER in intestinal epithelial cells and associated with ERS (42). TLR2 may induce enteritis by mediating IRE1α activation and chemokine production (42). The TLR2 signaling pathway is involved in LPS-induced ERS and may regulate the ATF4-CHOP pathway in response to ERS (43). Specifically, TLR2 activates IRE1α to produce XBP1, which induces transcription of IL-6 and TNF in macrophages to promote the production of inflammatory cytokines (44).

SELP, also named CD62 or P-selectin, is a glycoprotein that plays a crucial role in leukocyte recruitment, leukocyte rolling, and platelet adhesion. In the context of intestinal inflammation, mucosal microvascular endothelial cells overexpress cell adhesion molecules (CAMs) including P-selectin, ICAM1, and VCAM1 on both sides of the canal lumen to recruit leukocytes, which trigger a more severe inflammatory response. The study found that the expression of P-selectin, ICAM1, and VCAM1 was elevated in the inflamed colon tissue of individuals with inflammatory bowel disease (45). Inhibiting ERS has been shown to significantly reduce the expression of IL-6, ICAM-1, and VCAM-1 (46, 47). Furthermore, anti-VCAM1 antibody-coated mesenchymal stromal cells were found to attenuate experimental colitis via immunomodulation (48). PTPRC, also known as CD45, encodes a leukocyte antigen that regulates the immune response of T and B cells. PTPRC may promote the inflammatory response of atherosclerosis-related intracranial aneurysm by regulating immune lymphocytes (49). Increased numbers of CD45-positive cells in colon mucosa has been reported in colitis models (50), consistent with our result.

CCL4 belongs to the chemokine family and is known to attract inflammatory cells, including monocytes, macrophages, and lymphocytes to sites of inflammation. In addition, inhibiting CCL4 has been shown to reduce local inflammation and protect pancreatic β cells by increasing insulin expression in a type 1 DM animal model. Furthermore, inhibiting CCL4 with a specific antibody has been found to attenuate systemic inflammation, improve insulin resistance, and reduce circulating insulin levels in both type 2 diabetes animal models and metabolic syndrome animal models (51). CCL4 can activate PI3K/MAPK signaling and suppress ERS and inflammation by regulating UPR and NF-κB signaling proteins (52). In autoimmune diseases, studies have found a correlation between CCL4 polymorphisms and the risk of rheumatoid arthritis (RA). Modulating CCL4 expression could be a promising therapeutic strategy for treating RA (53). MMPs are a family of zinc-dependent endoproteases that play a role in regulating inflammatory and immune responses. During intestinal inflammation, MMPs drive mucosal damage. Our results show that MMP9 expression is significantly upregulated in inflamed intestinal mucosal regions.

Moreover, our iRegulon plugin analysis revealed that NFAT5, a homolog of NF-κB, and the calcineurin-activated NFATc transcription factors might regulate 11 ERSRGs. NFAT5 modulates various T-cell responses under different stress conditions and stimulatory contexts (54). Mice lacking NFAT5 specifically in T cells exhibited worsened intestinal pathology in an experimental colitis model, coupled with increased interferon gamma (IFNγ) messenger RNA (mRNA) in draining lymph nodes and colon (55). In individuals with lupus nephritis (LN), it has been observed that NFAT5 expression is increased, which is positively correlated with the expression of inflammatory cytokines and the severity of proteinuria (56). Furthermore, in a mice model of pristane-induced systemic lupus erythematosus (SLE), the absence of NFAT5 in myeloid cells prevented the development of LN and SLE (56). Activation of toll-like receptors (TLRs) that respond to damage-associated molecular patterns, induced the expression of NFAT5 in macrophages via stimulation of its promoter, which was essential for the intracellular signaling downstream of TLRs. NFAT5-deficient macrophages displayed elevated efferocytosis, and animals with myeloid deficiency of NFAT5 showed reduced Th1 and Th17 differentiation (56). However, the exact role of NFAT5 in ulcerative colitis pathogenesis requires further investigation of the complex interplay between NFAT5 and ERS-related genes.

To conclude, our study identified ERS-related molecular mechanisms in UC pathogenesis and novel molecular targets for the treatment of UC. We hope that our findings will provide new strategies for UC diagnosis and treatment. However, our study has limitations. First, it was based on bioinformatics analysis, and differences in microarray platforms, RNA extraction methods, and statistical methods could potentially bias the results. Second, we did not perform any *in vivo* or *in vitro* experiments for validation. Therefore, further research is necessary to provide convincing proof of our results.

## 5 Conclusion

We identified and validated 10 ERSRGs in UC. The effects of existing UC therapeutics on ERSRGs expression were explored. Moreover, new targets for small molecules targeting ERS in UC treatment were identified. The results suggest that ERS plays a vital role in UC pathogenesis, and noscapine may be a promising therapeutic agent for UC by affecting ERS.

## Data availability statement

These data were derived from the following resources available in the public domain: Gene Expression Omnibus (GEO) database (http://www.ncbi.nlm.nih.gov/geo). Further inquiries can be directed to the corresponding author.

## Author contributions

Conception and design: BD and YL. Administrative support: PH and SW. Provision of study materials or patients: BD and CL. Collection and assembly of data: BD and SW. Data analysis and interpretation: all authors. Manuscript writing: all authors. Financial Support: WD and FL. Optimization of the manuscript: BD and FL. All authors contributed to the article and approved the submitted version.

## Supplementary material

Supplementary Table 1 The list of endoplasmic reticulum stress-related genes.

Supplementary Table 2 The results of the Cmap dataset.
